# The Initial Defect in Weak-Sighted Children

**Published:** 1897-10-16

**Authors:** 


### THE INITIAL DEFECT IN WEAK-SIGHTED CHILDREN.

As a general thing it is only after being some time at school that a child's visual deficiencies are discovered, in other words, not until after the strain to which the eye has been exposed has led to the development of secondary troubles. It becomes a matter then of considerable interest to discover which is the most common initial lesion or congenital defect. According to Dr. Steiger of Berne, who has examined a large number of young children before secondary troubles had had time to develop, by far the major number were suffering from astigmatism. Much has been done lately in correcting myopia and hypermetropia in young children, but the suggestion is made that to go to the real root of the matter and to correct the original defect one must correct the initial astigmatism. Needless to say, in dealing with such young children one must use objective and not subjective tests.

